# Lucifer Yellow uptake by CHO cells exposed to magnetic and electric pulses

**DOI:** 10.2478/v10019-012-0014-2

**Published:** 2012-02-06

**Authors:** Leila Towhidi, Seyed Mohammad P Firoozabadi, Hossein Mozdarani, Damijan Miklavcic

**Affiliations:** 1 Tarbiat Modares University, Department of Medical Physics, Tehran, Iran; 2 Tarbiat Modares University, Department of Medical Genetics, Tehran, Iran; 3 University of Ljubljana, Faculty of Electrical Engineering, Ljubljana, Slovenia

**Keywords:** electroporation, electro-endocytosis, time-varying magnetic field, transmembrane molecular transport

## Abstract

**Background:**

The cell membrane acts as a barrier that hinders free entrance of most hydrophilic molecules into the cell. Due to numerous applications in medicine, biology and biotechnology, the introduction of impermeant molecules into biological cells has drawn considerable attention in the past years. One of the most famous methods in this field is electroporation, in which electric pulses with high intensity and short duration are applied to the cells. The aim of our study was to investigate the effect of time-varying magnetic field with different parameters on transmembrane molecular transport.

**Materials and methods.:**

‘Moreover, a comparison was made between the uptake results due to magnetic pulse exposure and electroporation mediated uptake.’ at the end of Background part. The Chinese hamster ovary (CHO) cells were exposed to magnetic pulses of 2.2 T peak strength and 250 μs duration delivered by Magstim stimulator and double 70 mm coil. Three different frequencies of 0.25, 1 and 10 Hz pulses with 112, 56 and 28 number of pulses were applied (altogether nine experimental groups) and Lucifer Yellow uptake was measured in each group. Moreover, maximum uptake of Lucifer Yellow obtained by magnetic pulses was compared to the measured uptake due to electroporation with typical parameters of 8 pulses of 100 μs, repetition frequency of 1 Hz and electric field intensities of 200 to 600 V/cm.

**Results and conclusions.:**

Our results show that time-varying magnetic field exposure increases transmembrane molecular transport and this uptake is greater for lower frequencies and larger number of pulses. Besides, the comparison shows that electroporation is more effective than pulsed magnetic field, but the observed uptake enhancement due to magnetic exposure is still considerable.

## Introduction

The cell membrane acts as a barrier that hinders free entrance of most hydrophilic molecules into the cell. Effects of electromagnetic fields on biological systems have been intensively investigated for possible damaging, diagnostic and therapeutic effects^1^–^3^ considering the cell membrane as the primary site of interaction.^4^ One of the interesting aspects of electromagnetic exposures is the incorporation of impermeant molecules such as macromolecules, drugs and proteins into biological cells without affecting cell physiological functioning and viability which can have numerous applications in medicine and biology. This has been mainly achieved, both *in vitro* and *in vi*vo by electroporation^5^–^8^, a process in which cells are exposed to a short duration (μs-ms) high intensity electric pulses (hundreds of V/cm).^8^–^10^ Electroporation is nowadays widely used in biotechnology^11^,^12^ and in the medical applications such as electrochemotherapy^13^–^16^ and gene electrotransfer.^5^,^17^–^21^ The suggested mechanism for this phenomenon is a structural change in plasma membrane resulting in pores formation.^6^,^22^ However, train of pulsed low electric field with a field strength value as low as 2.5–20 V/cm, frequency of a few hundred Hz and total exposure time of 1–10 min has been shown to be effective in enhancing uptake of large molecules into the cells. In this technique the pulse amplitude is not high enough to create pores and, assumingly, there are different pathways of molecular transport (*i.e.* electro-endocytosis).^23^–^25^ Furthermore, it was previously shown that the 900 MHz continuous sine wave and Global System for Mobile Communications (GSM) electromagnetic field increased *in vitro* molecular uptake by cells.^26^ The considered GSM were square pulses with a low frequency envelope of 217 Hz and high frequency carrier sine wave of 900 MHz. In that study it was also demonstrated that pulsed electric field of low intensity (2.6 V/cm), duration of 580 μs and with the frequency of the applied GSM (217 Hz) produced the same effect on LY uptake.^26^ In another study, the activation of K^+^ and Na+ pumping by an oscillating electric field (20 V/cm, 1 KHz) has been reported.^27^

Despite intensive studies on uptake increase due to different exposures, the possible effect of magnetic pulses on cell membrane permeability has not yet been tested. We hypothesize that pulsed magnetic field exposure, which induces electric field, will increase the molecular uptake of biological cells. In this paper, we present results of an experimental study of the effect of magnetic pulses on the cellular uptake. We determined the uptake of fluorescent dye Lucifer Yellow into adherent Chinese Hamster Ovary (CHO) cells due to time-varying magnetic field exposure. The uptake of fluorescent dye was determined for different frequencies and number of pulses. In addition, a comparison between the molecular uptake due to magnetic pulse exposure and the “conventional” electroporation was performed.

## Materials and methods

### Cells

Chinese hamster ovary cells (CHO-K1) (Pasteur Institute, Iran) were grown in HAM-F12 (Dulbecco’s modification of the Eagle’s Minimum Essential Medium – EMEM) containing 8% foetal calf serum, 160 μg/ml L-glutamine (all from Invitrogen-GIBCO BRL, Grand Island, NY, USA), 100 units/ml penicillin and 16 μg/mg gentamicin and incubated in 5% CO_2_ at 37°C. The cells were plated in 35 mm Petri dishes at 10^6^ cells per dish and incubated in HAM-F12 the day before the experiments. At the time of the experiments, the cells cover the surface of Petri dish completely in the form of monolayer. Just before applying the pulses, the culture medium in Petri dishes was replaced with 2 ml (2 mm media height in the dish) pulsing buffer (consisting of 250 mM sucrose, 10 mM KH_2_PO_4_/K_2_HPO_4_ and 1 mM MgCl_2_) containing 500 μM Lucifer Yellow (Invitrogen-Molecular Probes, Eugene, OR, USA).

### Magnetic pulse exposure

The magnetic pulse generator used in this study was Magstim (Magstim Rapid, Magstim Company, Withland, UK) which is usually used for non-invasive transcranial magnetic stimulation of human tissue.^28^–^30^ Such devices consist of a stimulating coil connected to a high-voltage discharge system which produces a very strong and short discharge current resulting in induced time varying magnetic fields. Based on Maxwell-Faraday equation, this strong pulsed magnetic field induces an electric field of the order of tens of volts per centimetre in the space around the coil.^31^,^32^

**FIGURE 1:**
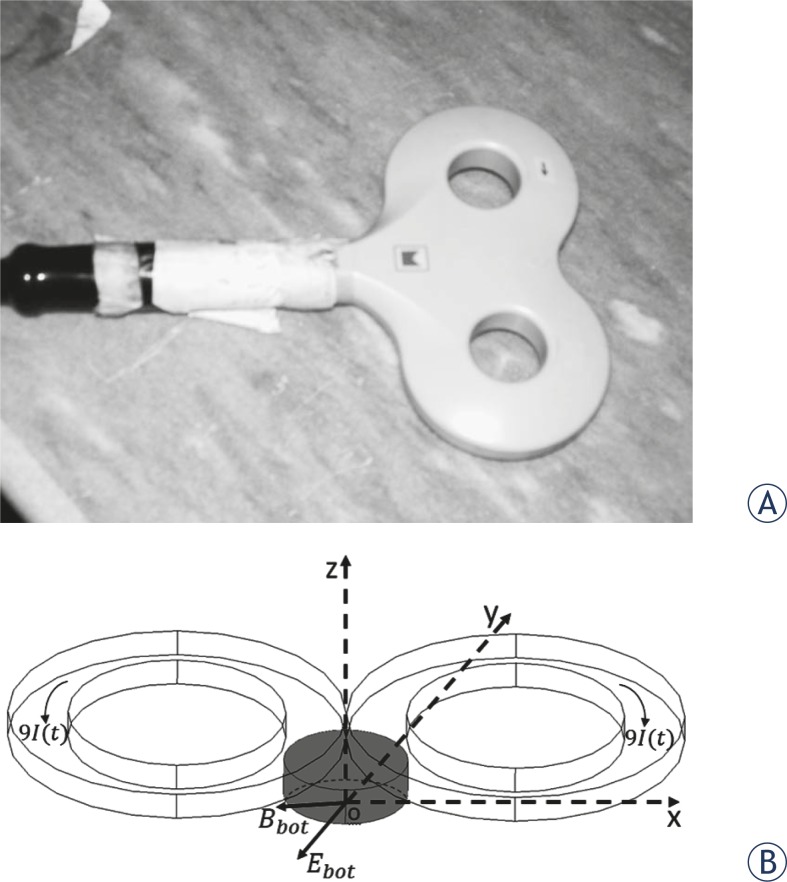
(A) Photograph of a 70 mm figure-of-eight coil used in the magnetic pulse exposure experiments. (B) Schematic of Petri dish location under figure-of-eight coil during magnetic field exposure. The direction of current passing through each winding is shown. The direction of resulted electric and magnetic field at the bottom of the Petri dish are displayed.

It has been demonstrated that figure-of-eight coils allow for a more focused and greater peak electric field than simple round coils.^31^,^33^ Moreover, the smaller coils induce higher magnetic field intensities than larger coils but the field falls off much more rapidly with distance.^31^,^33^ The optimum coil for our experiments in order to deliver the most intense fields with larger decay time constant was a 70 mm figure-of-eight coil with 100% energy transfer (Figure 1A). The magnetic field strength decreases rapidly with the distance from the stimulating coil so that the field strength is peaked close to the coil surface.^31^,^34^ Therefore, the Petri dishes containing the cells were placed under the coil where two windings meet and attached to the coil in order to expose cells to the strongest possible magnetic field. The geometry of the coils, the Petri dish in the experiments and direction of current flow through each winding of the coil are shown in Figure 1B.

During the exposures, the cells were attached at the bottom of the Petri dishes with radius of 17.5 mm and height of 10 mm. In previous studies, the distribution of magnetic and induced electric fields for different coils including the chosen coil in our experiments (70 mm figure-of-eight)^31^,^35^ are obtained. Considering results of these studies and the dimensions of the Petri dishes, the spatial distribution of field delivered to cells was approximately uniform at the Petri dish location. The strength and duration of pulses in all experiments were the same and investigated parameters were pulse repetition frequencies (0.25, 1 and 10 Hz) and number of pulses in each train of pulses (112, 56 and 28 pulses). The main frequency considered in this study was 0.25 Hz in order to have several minutes exposure time before warming up the device and the coil. In this frequency, the maximum possible number of delivered pulses was about 112. Then, to demonstrate the effect of number of pulses, half and a quarter of 112 (*i.e.* 56 and 28 pulses) for each pulse repetition frequency were also studied.

### Electric pulse exposure

A pair of parallel Pt/Ir wire electrodes with 0.8 mm diameter and 25 mm length spaced 5 mm from each other was positioned at the bottom of Petri dishes. Electric pulses were generated by a Cliniporator device (IGEA s.r.l., Carpi, Modena, Italy). The electroporation results depend on pulse parameters such as pulse shape, frequency, duration and number of pulses.^8^,^10^,^35^ We have chosen some typical electroporation parameters of 8 pulses of 100 μs and repetition frequency of 1 Hz. Based on previous electroporation studies on suspension and attached biological cells^10^,^36^,^37^, the voltages delivered to the electrodes were selected to be between 100 and 300 V (*i.e.* voltage-to-distance ratios of 200 to 600 V/cm) with increment of 50 V (*i.e.* voltage-to-distance ratio incrementing by 100 V/cm).

### Determination of Lucifer Yellow uptake

Lucifer yellow (LY) is an impermeant florescent dye^38^ which in case of penetrating through the membrane, stays inside the cell and does not affect the cell viability due to its non-toxicity.^39^ Thus using a standard protocol^40^, the quantity of Lucifer Yellow taken up by the cells can be measured at given time after the exposure of cells to magnetic or electric pulses. In our experiments, after the exposure of cells to the magnetic or electric pulses, the cells were incubated at room temperature for 40 minutes to allow resealing of the plasma membrane.^8^,^41^ The cells were then washed four times with phosphate buffer saline (PBS, Life Technologies, Paisley, UK) to remove Lucifer Yellow from extra-cellular medium. Cells were then broken down by adding diluted HCl for 12 hours and then the total fluorescence taken up by the cells was measured in arbitrary units on a spectrofluorometer (Shimadzu RF-5000, Japan). The excitation and emission wavelengths were set at 418 and 525 nm, respectively.

As the electroporation electric pulses were applied to the cells via two electrodes separated 5 mm and positioned at the bottom of the Petri dishes, only the cells located between these two electrodes are exposed to the electric field (25×5 mm^2^). But in the case of time-varying magnetic field exposure, the magnetic pulses are applied to all the cells in the Petri dish located under the coil (π×35^2^/4 mm^2^). Thus to make the obtained data comparable, the ratio of exposed area was taken into account. This has been accomplished via multiplying the measured fluorescence with the ratio of the Petri dish area and the area between two electrodes.

The laboratory temperature during the experiments was about 25°C. Results were given as a percent of control. The procedures for the control group samples were identical to exposed cells (*i.e*. Lucifer Yellow was added to their medium) except that no pulses were delivered. All results are given as average of 4 to 13 repetitions and are presented in bar graphs. In order to perform a statistical analysis, Mann-Whitney Rank-Sum test was used.

## Results

**FIGURE 2:**
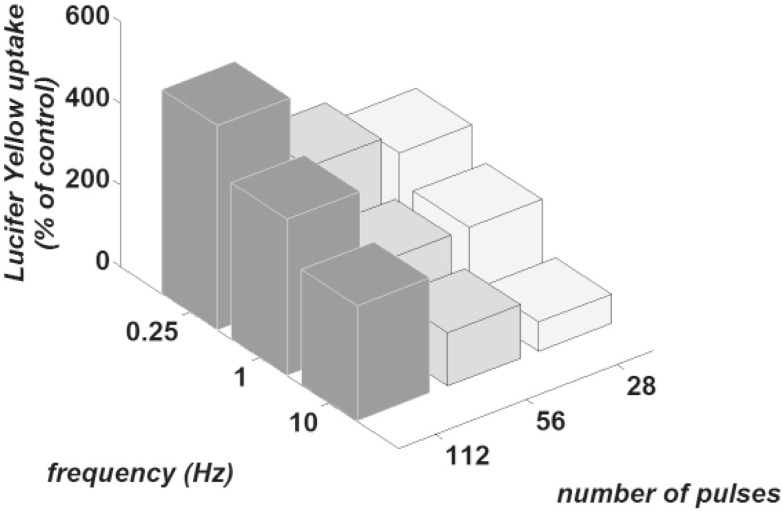
Dye uptake of attached CHO cells for three different frequencies 0.25, 1 and 10 Hz with three different numbers of pulses 112, 56 and 28 for each chosen frequency. Attributed number to the control group was chosen 100 and the fluorescence of other groups was computed as the percent of control fluorescence.

Figure 2 shows the uptake of Lucifer Yellow for 9 different sets of parameters of magnetic field exposure (three different frequencies of 0.25, 1 and 10 Hz pulses for 112, 56 and 28 pulses). The value of the control group fluorescence was considered as 100 and the fluorescence of experimental groups was normalized to the control group. The comparisons of the uptake between the control group and exposed groups and also between exposed groups were performed using the Mann-Whitney Rank-Sum test. The P-value for the significance level of distinct groups was set equal to 0.05.

The statistical results show that for all frequencies of 0.25, 1 and 10 Hz, exposure of 112 pulses is significantly more efficient in comparison with a control group, 56 and 28 pulses. Moreover, for none of the 0.25, 1 and 10 Hz frequencies, one can observe any significant difference between 28 and 56 pulses groups. For 10 Hz group, there is even no significant difference between the last two groups and the control group.

Exposure to 112 of pulses and frequency of 0.25 Hz result in the highest uptake of Lucifer Yellow. The same number of pulses delivered at frequencies of 1 and 10 Hz shows, however, no significant difference. With 56 pulses, 0.25 Hz frequency is more effective than the two other frequencies while frequency of 10 Hz shows no significant difference with the control group. With 28 number of pulses, 0.25 and 1 Hz make no significant difference at 28 pulses, but they are more efficient relative to the control group; between 10 Hz group and the control group, however, no difference was observed.

Based on above observations, we may state that the dye uptake is greater for lower frequencies and larger number of pulses.

**FIGURE 3:**
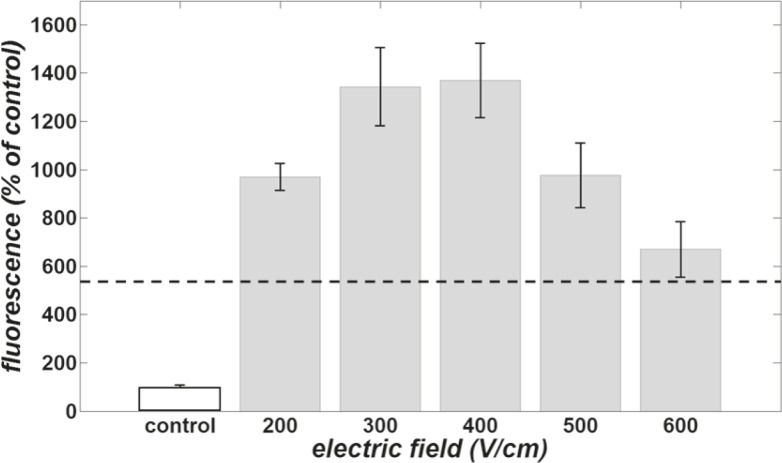
Dye uptake of attached CHO cells. The white bar shows the control group normalized to 100. The fluorescence of other groups was computed as the percent of control fluorescence. The gray bars show uptake attributed to different electric field exposure amplitudes of 8 pulses of 100 μs and 1 Hz for electroporation experiments. The dashed line demonstrates the greatest uptake of cells due to magnetic field exposure (112 pulses of 0.25 Hz). Vertical bars represent standard deviation of the mean.

In order to compare cell exposure to magnetic pulses with the conventional method for membrane permeability increase (*i.e.* electroporation), the uptake enhancement of attached CHO cells due to determined applied electric pulses were also investigated. The cells were exposed to 8 pulses of 100 μs duration and 1 Hz pulse repetition frequency of five different electric field intensities (200 to 600 V/cm with 100 V/cm increment). The resulted value for cellular uptake of LY due to electroporation with different pulse amplitudes are displayed in gray in Figure 3. For an easier comparison, the highest measured fluorescence of cells due to the magnetic pulse exposure (112 pulses of 0.25 Hz) is shown with dashed line. The control group is illustrated in white. The comparisons of the uptake between the control group, groups exposed to electric pulses and groups exposed to magnetic pulses were performed using the Mann-Whitney Rank-Sum test. The results of this comparison show that with selected parameters of electric and magnetic exposures, both give rises to the uptake enhancement and have a significant difference with the control group. Although the results show that all the electric pulse exposure groups, except the one with 600 V/cm field strength, were significantly more effective than magnetic pulse exposure groups (Figure 3).

## Discussion

The results of the experimental study on exposing the cells by magnetic pulses show that magnetic pulses can efficiently increase Lucifer Yellow up-take by CHO cells. The amount of florescence measured for the control group not exposed to the pulses was ascribed to the remaining extracellular Lucifer Yellow after washings and also to the uptake due to normal endocytotic process. The results show that all experimental groups except groups exposed to 28 and 56 pulses at 10 Hz have the significantly higher uptake of Lucifer Yellow when compared to the control group. We thus conclude that generally applying magnetic pulses can enhance the uptake of molecules by cells. The results show (Figure 2) that magnetic pulses of the same number but different frequencies to the cells indicate that lower frequencies are more efficient. Furthermore, we observe that increasing the number of pulses enhances dye uptake. It is important to note that the total exposure time for different numbers of pulses used in experiments was different for different frequencies. For example total exposure time for 112 pulses of 0.25, 1 and 10 Hz frequencies were 467, 116 and 11 seconds, respectively.

In addition, the uptake of Lucifer Yellow due to exposure to magnetic pulses of 112 pulses of 0.25 Hz was compared to the exposure of cells electro-porated by electric pulses of 1 Hz, duration of 100 μs and electric field amplitudes usually used in electrochemotherapy protocols. The results of this comparison show that with the selected parameters of exposures, both give rises to the uptake enhancement and have a significant difference with the control group, although increase due to exposure of cells to magnetic field is considerably smaller when compared to “classical” electroporation (Figure 3).

The increased permeability in electroporation is believed to be due to exceeding of induced trans-membrane voltage from a critical value of few hundred mV. Note that a transmembrane voltage of this amplitude can be by exposing cells to an electric field of few hundred V/cm.^42^,^43^ This high electric field, as suggested in literature, causes structural changes and pore formation in the cell membrane which in turn give rise to an increase of permeability and cellular uptake.^6^ In magnetic pulse exposure, the induced electric field due to a time varying magnetic field at the cells position was about 6 V/cm.^31^ This field strength is by far much smaller than the electric field needed for electroporation^44^ and is unable to form pores in the plasma membrane (although the exposure time in the latter is by far much larger *i.e.* 7 minutes in the latter, contrast to 8 seconds in the former). Therefore, we need to seek an alternative mechanism responsible for the observed increase of dye uptake by the cells in our experiment using magnetic pulses.

Previous studies have shown that long train of low pulsed electric field with a field strength value as low as 2.5–20 V/cm, frequency of a few hundred Hz and total exposure time of 1–10 minutes enhances the uptake of large molecules into the cells.^24^,^25^ The reason for the uptake increase was explained by the imbalance of charge distribution in the two opposite leaflets of the cell membrane due to the electric forces. This charge imbalance was stipulated to stimulate endocytotic-like process named electro-endocytosis. The efficiency of incorporation of macromolecules into the cells depended on the electrical parameters of exposure such as pulse amplitude, duration, frequency and total time of exposure.^25^ In another study, the effect of mobile phone electromagnetic fields with envelope frequency of 217 Hz, carrier frequency of 900 MHz and pulse duration of 580 μs were investigated and the uptake increase was reported.^26^ Furthermore, it was also demonstrated that the electric component of electromagnetic fields was responsible for this increase. The associated pulsed electric field in their study featured low intensity electric field 1.2–8 V/cm, pulse duration from 75 to 580 μs, frequency from 50 to 400 Hz and total exposure duration from 5 to 90 min.^26^ It was demonstrated that the dye uptake in both cases increased due to fluid-phase endocytosis. The exposure of cells to such electric fields results in mV range induced transmembrane voltage – similar to studies reporting electro-endocytosis. In other studies on electroporation, it was proposed that the tangentional component of the field on the external leaflet of the membrane may result in electrophoretic mobility of the charges, proteins and lipids of the membrane, enzyme fluctuation or ruffling. This in turn may induce endocytosis which might last for about one hour after applying pulses.^45^–^48^

We now discuss the case of magnetic pulse exposure. Pulsed magnetic field induces an electric field. This electric field is circular and tangentional in the interacellular region. The order of the electric field is comparable with low amplitude electric field in foregoing studies. Thus, the attributed cellular uptake increase can be convincingly explained based on the suggested above mentioned mechanisms with exerting electrophoretic mobility on the outer leaflet of the membrane and inducing endocytosis. We, thus, suggest that electro-endocytosis might facilitate the passage of external substances into the cell.

On the other hand, it is suggested in a survey that magnetic field pulses might create metastable cell membrane pores via interaction with membrane-attached magnetic particles and ubiquitous ferromagnetic contaminant particles exist in solutions and media.^49^ Therefore, another possible mechanism for the uptake enhancement due to magnetic pulse exposure is the interaction of transient magnetic field with these particles. This may cause some effects even creating membrane pores due to rotational motion of a membrane-bound particle and transferring enough energy and consequently increasing the cellular uptake.

The purpose of our study was to test enhanced molecular uptake by cells due to the exposure of cells to magnetic pulses. According to the results of our study, with applying time-varying magnetic field the uptake of extracellular molecules to the cells increases significantly. Our results show that this increase is more obvious for lower frequencies and larger number of pulses (also associated with longer time exposure). We also give plausible explanations of the underlying mechanisms. It remains, however, to determine exact mechanisms of this increased uptake of molecules and to test if this technique can be used also *in vivo* for example for the treatment of tumours like electroporation in electrochemotherapy. Considering the fact that magnetic fields *i.e.* transcranial magnetic stimulation are able to focus and pass unhindered through skin, muscle and bone, this approach can potentially be useful in treating deep-seated tumours noninvasively.
